# Cytokine-Mediated Induction and Regulation of Tissue Damage During Cytomegalovirus Infection

**DOI:** 10.3389/fimmu.2019.00078

**Published:** 2019-01-29

**Authors:** Mathew Clement, Ian R. Humphreys

**Affiliations:** Division of Infection and Immunity/Systems Immunity University Research Institute, Cardiff, United Kingdom

**Keywords:** cytokine, cytomegalovirus infection, immunopathologic process, virus, mcmv

## Abstract

Human cytomegalovirus (HCMV) is a β-herpesvirus with high sero-prevalence within the human population. Primary HCMV infection and life-long carriage are typically asymptomatic. However, HCMV is implicated in exacerbation of chronic conditions and associated damage in individuals with intact immune systems. Furthermore, HCMV is a significant cause of morbidity and mortality in the immunologically immature and immune-compromised where disease is associated with tissue damage. Infection-induced inflammation, including robust cytokine responses, is a key component of pathologies associated with many viruses. Despite encoding a large number of immune-evasion genes, HCMV also triggers the induction of inflammatory cytokine responses during infection. Thus, understanding how cytokines contribute to CMV-induced pathologies and the mechanisms through which they are regulated may inform clinical management of disease. Herein, we discuss our current understanding based on clinical observation and *in vivo* modeling of disease of the role that cytokines play in CMV pathogenesis. Specifically, in the context of the different tissues and organs in which CMV replicates, we give a broad overview of the beneficial and adverse effects that cytokines have during infection and describe how cytokine-mediated tissue damage is regulated. We discuss the implications of findings derived from mice and humans for therapeutic intervention strategies and our understanding of how host genetics may influence the outcome of CMV infections.

## Introduction

Human cytomegalovirus (HCMV) is a ubiquitous beta-herpesvirus that has co-evolved with its host for millions of years and acquired multiple immune evasion functions that manipulate and hide the virus from host immunity (1, 2). Primary HCMV infection and latency in immune-competent hosts is usually asymptomatic (3). Thus, HCMV is typically thought to establish lifelong infection without inducing overt pathology often triggered by other viruses. It is becomingly apparent, however, that chronic HCMV carriage in ‘healthy individuals’ may exacerbate conditions from general frailty (4) to cardiovascular disease (5).

HCMV causes morbidity and mortality in immune-compromised patients including transplant recipients and HIV co-infected individuals. Solid-state organ or human stem cell transplantation remains challenging as immune suppression can facilitate uncontrolled HCMV reactivation from host and/or donor tissue, resulting in organ pathology and systemic disease (6). HCMV co-infection is the leading cause of vision loss in untreated HIV/AIDS individuals (7, 8) and remains an issue in patients receiving anti-retroviral therapy (9). HCMV causes gastrointestinal and neurological diseases during HIV co-infection (7, 10). Further examples of viral-induced morbidity include congenital infection where HCMV is the leading infectious cause of all congenital birth defects (11, 12). Life-long neurological defects ensue, including microcephaly, encephalitis, seizures, and blindness, and HCMV is the leading cause of congenital deafness (6, 12, 13).

The fact that HCMV preferentially causes disease in immune compromised individuals highlights the importance of immune control of virus replication. Indeed, many HCMV-associated disease manifestations correlate with viral replication and respond to antiviral drug treatment. However, certain syndromes, particularly chronic diseases, do not typically correlate with high HCMV load (14), suggesting that direct cellular destruction by virus is not the sole cause of tissue damage.

**Figure 1:**
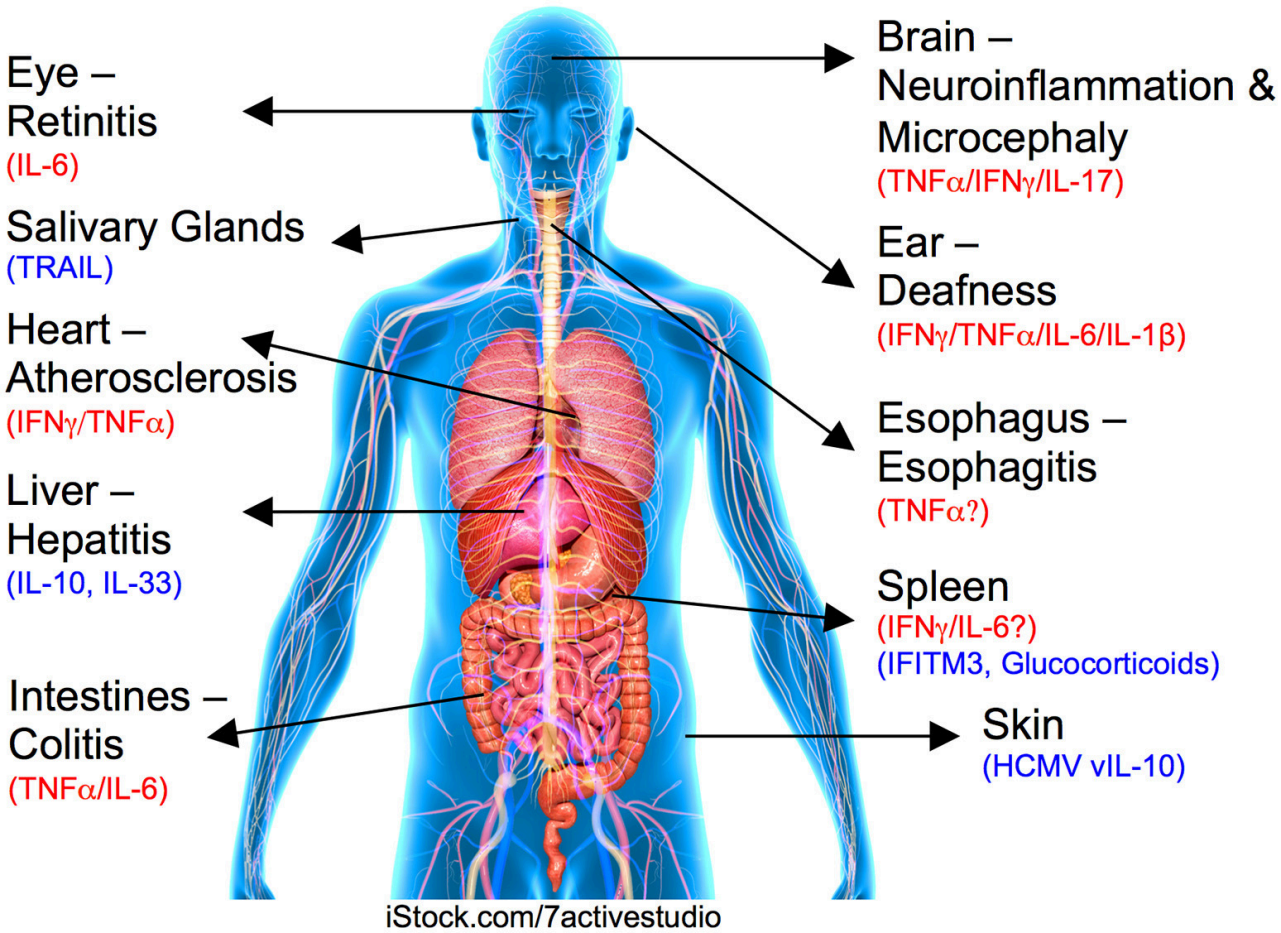
Role for cytokines in CMV-induced organ pathologies. Organ or tissue and associated CMV-induced disease is labeled with inflammatory pathogenic cytokines highlighted in red and regulatory/suppressive pathways shown in blue.

Cytokines participate in immune responses to viruses that activate innate immune responses and orchestrate the development of adaptive antiviral immunity. However, uncontrolled cytokine production can cause off-target effects, participating in various immune-driven pathological processes. Due to the limitations of what can be investigated in humans, the murine CMV (MCMV) model has been used for decades to study mechanisms influencing CMV pathogenesis *in vivo*, including how cytokines orchestrate antiviral immunity [summarized in detail elsewhere (15)]. Herein, we examine evidence from both clinical studies and experimental models of CMV infection showing that although cytokines are required to limit viral replication, they can cause host damage. We discuss these findings in the context of different tissues where damage during CMV infection can ensue and describe the mechanisms that restrict these harmful processes (see Figure 1 for summary).

## Pro-Inflammatory cytokines, Systemic Cytomegalovirus-Induced Disease, and Organ Damage

Cytokine responses during HCMV viremia have been mostly studied in the transplantation setting where time of virus exposure is known. Following initial replication, sustained type 1 cytokine signatures are observed that are characterized by production of IFNγ [in some but not all studies 16], IL-18 and IL-6, and is further accompanied by acute phase protein and chemokine (IP-10) secretion (16, 17). T-cells are implicated as a significant source of type 1 cytokines (18, 19). Furthermore, numerous pro-inflammatory chemokines and cytokines, including IL-6, are secreted directly following HCMV infection (20). HCMV triggers cytokine production through the stimulation of pattern recognition receptors (PRRs), most notably Toll-like receptor 2 (21), the cytoplasmic DNA sensor STING (22) and IFI16 (23). Mice defective in PRRs mount reduced cytokine responses to MCMV *in vivo* (24–26). Although differences in the relative contributions of PRRs to the recognition of MCMV and HCMV may exist, these data suggest that innate immune recognition of viral infection by PRRs contributes to HCMV-induced cytokine profiles. Furthermore, *in vitro*, HCMV stimulation of peripheral blood-derived monocytes increases expression of TLRs, CD14, and adaptor molecules and transcription factors downstream of TLRs (27). Thus, active HCMV replication likely induces systemic pro-inflammatory cytokine responses both following via direct host recognition but also, potentially, by priming the host immune response to respond strongly to unrelated microbial signals.

Given the established role for type 1 cytokines in antiviral immunity, is such a response to CMV infection a bad thing for the host? Certainly, substantial evidence from clinical and experimental studies point toward a protective role for type 1 cytokine responses in cytomegalovirus infections (28–30). However, studies using MCMV show that T-cell responses, particularly CD8^+^ T-cells, known to be induced by type 1 cytokines cause substantial tissue damage if insufficiently regulated (31, 32). Also, severe inflammatory cytokine responses or “cytokine storms” occur during MCMV hepatitis (33). Thus, these processes may drive acute HCMV-associated diseases. Furthermore, HCMV is implicated in organ rejection (34, 35) and, in cardiac transplants, graft atherosclerosis (36). Experimental studies using MCMV have recapitulated the observation that acute infection and viral reactivation can influence graft longevity (37, 38). MCMV reactivation induces expression within the graft of IFNα and IL-12 (37), implying that viral infection may elicit cytokine responses that activate cellular immunity capable of mediating graft rejection. Furthermore, HCMV induces IP-10 and fractalkine production during infection (17, 39), both of which are markers of allograft rejection (40).

HCMV establishes life-long infection within multiple host tissues (41) where some genomes are silent but others are transcriptionally active and express many genes (41–43). Immunological data highlights the likelihood that frequent reactivation events occur that re-stimulate the host immune system (44). Subsequently, HCMV may contribute to cytokine mediated inflammatory diseases in latently-infected immune competent individuals via continued gene transcription and reactivation, stimulating immune recognition and subsequent cytokine production. For example, HCMV is implicated in cardiac diseases (45) including atherosclerosis (46) where plaque formation and instability is an inflammatory-driven processes initiated by IFNγ (47). HCMV also induces accumulation of virus-specific cytotoxic CD4^+^ T-cells expressing CX3CR1 (48). CX3CR1 binds fractalkine which is expressed by activated endothelium in response to TNFα and IFNγ produced by HCMV-specific T-cells (39). Interestingly, the HCMV chemokine receptor homolog US28 also binds fractalkine (49) and may contribute to localized inflammation. Thus, HCMV-induced cytokine and T-cell responses may mediate endothelial damage that in turn promotes vascular diseases and contributes to damage in multiple tissues and organs. Whether such processes underpin other harmful associations of HCMV, such as increased frailty in elderly individuals (4), is unclear.

Cytokines may also indirectly enhance tissue damage by promoting CMV reactivation and subsequent replication. IL-6 promotes HCMV reactivation in dendritic cells via ERK-MAPK mediated transcriptional induction of major immediate early (IE) genes (50, 51). TNFα and IL-1β also induce IE gene transcription by latent HCMV (52–54) and are implicated in reactivation of HCMV and/or MCMV *in vitro* and *in vivo* (55–59). An additional role for IFNγ in initiating HCMV reactivation has been described (56, 58). Data from MCMV suggest that overt pro-inflammatory cytokine responses may also impinge on innate antiviral immunity. Inadequate pro-inflammatory cytokine regulation can promote activation-induced NK cell death (60, 61) in a process involving IL-6 (60). Thus, inflammatory cytokines may directly and indirectly promote virus replication, which in turn drives peripheral tissue damage.

## Cytokines and Damage in Immune Privileged Sites

When HCMV accesses immune privileged organs, immune-mediated pathology can ensue. HCMV-induced retinitis is a significant problem in AIDS/HIV patients (7–9). Interestingly, elevated expression of type 1 cytokines including IL-6 and IFNγ in aqueous and/or vitreous fluids from patients is detectable (62–64). Systemic CMV infection in immune competent mice induces significant myeloid cell and T-cell infiltrations into ocular tissue including the neural retina (65). Although cytokines likely play a role in mediating these inflammatory processes in immune competent hosts, this has yet to be investigated.

A role for inflammation in HCMV-induced hearing loss in infants is suggested by autopsies showing inner ear inflammation (66, 67). In mice, systemic infection of newborns induces progressive hearing loss and decreased spiral ganglia neuron density that is indicative of congenital HCMV infection (68). In MCMV, hearing loss does not correlate with the presence of virus in the cochlea but rather associates with persistent expression of chemokines and pro-inflammatory cytokines including TNFα, IL-6, and IL-1β (68). Similarly, intracranial MCMV infection induces hearing loss and chronic inflammatory cytokine expression (69).

Murine neonatal infection models have also been used to recapitulate central nervous system pathology triggered by congenital HCMV infection. After systemic infection, MCMV induces widespread focal encephalitis accompanied by mononuclear inflammation and microglial activation (70, 71), including TNFα expression (72). This is accompanied by STAT1 activation and IFN (type I and II) expression, in addition to TNFα (73). Interestingly, glucocorticoid treatment of these mice reduced cytokine expression and associated morphogenic abnormalities and cellular inflammation without influencing virus load, suggesting that virus-induced inflammation could be safely targeted to improve CMV-induced CNS pathogenesis (73). Indeed, neutralization of TNFα reduced expression of cytokines and myeloid cell activation and accumulation in the brain, and corrected cerebellar abnormalities and developmental gene expression (74). These important studies provide proof-of-concept that anti-inflammatory approaches can be safely utilized to ameliorate CMV pathogenesis *in vivo*.

HCMV is implicated in esophagitis in HIV-infected individuals and associates with elevated TNFα production (75). Inflammatory bowel diseases are common during HIV co-infection (7) and HCMV maintains active replication in the gastrointestinal epithelium of individuals treated with antiretroviral therapy, where replication disrupts epithelial integrity in a manner partially dependent upon IL-6 (76). HCMV also associates with gastrointestinal inflammation in healthy individuals (77), where the virus may drive local production of cytokines such as TNFα (78) via induction of pattern recognition receptor expression and/or downstream, adaptor molecules (27, 79).

HCMV may also impact on neurological diseases in adults, with associations with HIV-associated neurological disorder (HAND) and impaired cognitive performance in HIV-infected individuals being reported [reviewed in (10)]. The link between HCMV and multiple sclerosis in immune competent hosts is controversial, with contradicting findings regarding the association between HCMV seropositivity and disease occurrence (80–82). In the murine experimental autoimmune encephalomyelitis (EAE) experimental model, MCMV worsens disease in genetically susceptible mice (83) and increases EAE occurrence in resistant (BALB/c) strains. Here, infection increases CD4 T-cell-dependent disease that is associated with IFNγ- and IL-17-expressing T-cells (84), further demonstrating that CMV can exacerbate tissue damage in the central nervous system.

Like many herpesviruses, HCMV is implicated as a risk factor in Alzheimer's Disease (AD) and cognitive decline (85). PBMCs from HCMV seropositive AD subjects produce more IFNγ following polyclonal and viral protein stimulation than non-AD subjects (86), and IFNγ is detectable only in cerebrospinal fluid of HCMV seropositive but not seronegative AD patients (87). Thus, although the role of HCMV in AD development is controversial, (88) it appears that HCMV-infected AD sufferers exhibit heightened cytokine responses which in turn could contribute to disease development and/or progression.

## Regulation of Cytokine-Driven CMV-Induced Pathogenesis

Despite its inflammatory potential, HCMV rarely causes inflammatory conditions in healthy individuals. Furthermore, infection in immune compromised and immunologically immature hosts does not always cause overt tissue damage, suggesting that virus-induced inflammatory cytokine responses are tightly regulated.

### Regulatory T-Cells

The association between inducible regulatory T-cell (iTregs) expansions and reduced vascular pathology in elderly HCMV-infected individuals suggests a protective function for Tregs in HCMV infection (89). In MCMV, Tregs (promoted by IL-33) restrict liver pathology following systemic MCMV infection (32) and chronic reactive gliosis triggered by MCMV encephalitis (90). Although hepatic Tregs are known to be dependent upon IL-33 (32), whether Treg-mediated control of pathogenic T-cell responses involves restriction of inflammatory cytokine secretion is currently unknown.

### Cytokines

Inflammatory cytokine responses during acute HCMV infection are accompanied by secretion of the immune modulatory cytokine IL-10 (16, 91). HCMV re-programmes human hematopoietic progenitor cells (HPCs) into immune-suppressive monocytes that express IL-10 in a process requiring US28 (92). In mice, genetic and pharmacological targeting of IL-10 demonstrates that IL-10 limits systemic inflammatory cytokine responses induced by CMV, including IL-6 and TNFα (61, 93, 94). This alleviates MCMV-induced disease, assessed using body weight (93, 94), and weight loss in IL-10^−/−^ mice is alleviated by TNFα neutralization (93). IL-10 also restricts MCMV-induced hepatic inflammation and preserves liver function by limiting inflammatory effector cell infiltration, hepatocyte apoptosis and necrosis (95, 96). Experiments performed in perforin-deficient mice that are unable to control MCMV replication reveal that IL-10 restricts liver inflammation primarily by limiting pathogenic CD8^+^ T-cell responses (31), a conclusion supported by data derived from immune competent Il-10^−/−^ mice (95). Following injection of MCMV into the brain, IL-10 limits fatal immunopathology characterized by pro-inflammatory cytokine production and neutrophil infiltration (97, 98). Although the physiological relevance of some of these experiments in terms of HCMV pathogenesis is unclear, these data clearly highlight that IL-10R signaling can suppress CMV-induced immune pathology.

Importantly, genetic variation within the human IL-10 gene correlates with altered HCMV disease occurrence following allogeneic stem cell transplantation (99) and during HIV co-infection (100). This suggests that host genetic variation may influence tissue damage caused by HCMV-induced cytokines. Furthermore, HCMV encodes a functional IL-10 otholog (UL111A, vIL-10) that is expressed in lytic replication (101) and an alternate isoform in latency [LAcmvIL-10 102]. vIL-10 suppresses numerous innate and adaptive host immune responses including pro-inflammatory cytokine secretion (103, 104). Given that cellular IL-10 promotes MCMV carriage (93, 105–107), one may predict that HCMV vIL-10 facilitates virus persistence. However, using rhesus macaque CMV (rhCMV) that, like HCMV but not MCMV, expresses *UL111A*, it has been demonstrated that vIL-10 restricts acute inflammation at the initial site of infection, the skin. Interestingly, UL111A had no obvious impact on virus shedding in these experiments. This implies that virus persistence may not be influenced by UL111A *in vivo* (108) but instead that restriction of tissue pathology is an important function of viral IL-10 orthologs and perhaps other immune evasion gene products expressed by HCMV. Intriguingly, certain clinically-isolated HCMV strains have disrupted UL111A genes (109, 110). It will be interesting to investigate whether these HCMV strains preferentially associate with overt inflammatory responses.

IL-27 is an IL-12 family member that restricts numerous infection-induced pathologies (111). IL-27 facilitates MCMV persistence in the mucosa by suppressing IFNγ^+^ (107) and/or cytotoxic (112) CD4^+^ T-cells. Given that cytotoxic CD4^+^ T-cells are implicated in tissue damage (113), IL-27-faciliated shedding of virions may be a necessary evil to restrict the development of these cells. Data regarding the function of IL-27 during HCMV infection is limited. Spector and colleagues identified that IL-27 limits IFNγ expression by virus-specific T-cells in HIV^+^ and HIV^−^ HCMV-infected individuals. This was accompanied by IL-27-mediated induction of IL-10 secreting CD4^+^ T-cells (114). Whether IL-27 also alters the development of HCMV-specific cytotoxic T-cells is unknown. However, overall these data are consistent with the idea that IL-27 restricts chronic tissue damage by limiting HCMV-specific T-cell responses.

Data from HCMV and MCMV highlights that the cytokine TNF-related apoptosis-inducing ligand (TRAIL) contributes to control of virus replication (115–117). During persistent MCMV infection in the salivary glands, however, TRAIL expression by NK cells restricts pathogenic CD4^+^ T-cell responses in this tissue. TRAIL-deficient mice exhibit hallmarks of Sjogren's syndrome (SS), an autoimmune disease of the salivary glands that is characterized by ectopic germinal center-like structures in the glands, elevated autoantibody production and impaired saliva secretion (113). Thus, TRAIL can limit both viral replication and potentially harmful infection-induced inflammatory responses.

### Antiviral Restriction Factors

Interferon induced transmembrane protein 3 (IFITM3) is an antiviral restriction factor that inhibits endocytosis-dependent cell entry of numerous viruses (118). IFITM3 polymorphisms associated with reduced function are linked to increased risk of severe viral pathogenesis, most notably influenza-induced disease (119–121). Although IFITM3 does not directly impinge on either MCMV or HCMV replication (60, 122), *Ifitm3*^−/−^ mice are dramatically more susceptible to MCMV-driven pathogenesis (60). Disease, which can be fatal, consists of extensive weight loss, transient pulmonary and hepatic mononuclear inflammation, and extensive and irreversible splenic damage. Blocking the action of IL-6 alleviates pathogenesis in MCMV-infected *Ifitm3*^−/−^ mice and also inhibits activation-induced NK cell death and promotes NK cell immunity (60). Thus, it is unclear whether IL-6 drives CMV-induced pathology by promoting tissue damage and/or by impairing cellular antiviral innate immune responses and subsequent control of virus replication. Irrespective, these data again highlight the possible role for genetics in determining host cytokine responsiveness to HCMV and the subsequent disease outcome.

### Glucocorticoids

Endogenous glucocorticoids are steroid hormones produced in the adrenal cortex following activation of the hypothalamic-pituitary-adrenal (HPA) axis. Initial inflammatory cytokine responses during acute MCMV infection are accompanied by robust glucocorticoid production (123, 124), the maximal release of which is dependent upon virus-induced IL-6 (123). The importance of glucocorticoids in modulating CMV-induced pathogenesis is highlighted in studies where mice are rendered globally deficient in glucocorticoids by adrenalectomy and display increased production of pro-inflammatory cytokines and susceptibility to TNFα-mediated lethal disease (125). Furthermore, glucocorticoid receptor signaling in NK cells, via an axis involving the inhibitory PD-1 receptor, exerts tissue-specific regulation of IFNγ production. Here, unrestricted NK cell expression of IFNγ in spleens of mice lacking the glucocorticoid receptor in NCR1^+^ cells results in necrotizing splenitis and destruction of the white pulp (124). Although pathology in medically important sites of CMV pathogenesis like the liver were unaffected by this process (124), these data suggest that neuro-immune pathways may be critical for control of cytokine-driven pathogenesis during CMV infection.

## Conclusions

Many associations exist between production of inflammatory cytokines and CMV-associated pathologies in humans and in experimental systems. Experimental models like MCMV have their limitations in terms of variations in virus genetics (including lacking key immune regulatory genes like vIL-10) and the imperfect recreation in mice of HCMV-induced pathologies. However, important predictions regarding roles that cytokines play in virus-induced tissue damage and how inflammatory cytokines are regulated can be derived from these studies. Moving forward, these models will be critical to examine whether targeting CMV-induced inflammation is an effective, safe and viable approach to alleviating pathogenesis. Understanding exactly how cytokines cause tissue damage and how production of these cytokines is regulated will hopefully lead to more refined and effective strategies to help alleviate the pathological consequences of HCMV infection. These studies may also help identify host genetic variations that influence cytokine responsiveness and susceptibility to HCMV disease. Finally, these studies may help form novel hypotheses regarding the possible influence of genetic variation in virus-encoded immune evasion genes on HCMV pathogenesis.

## Author Contributions

IRH defined the manuscript focus and structure. MC and IRH wrote and edited the manuscript.

### Conflict of Interest Statement

The authors declare that the research was conducted in the absence of any commercial or financial relationships that could be construed as a potential conflict of interest.
